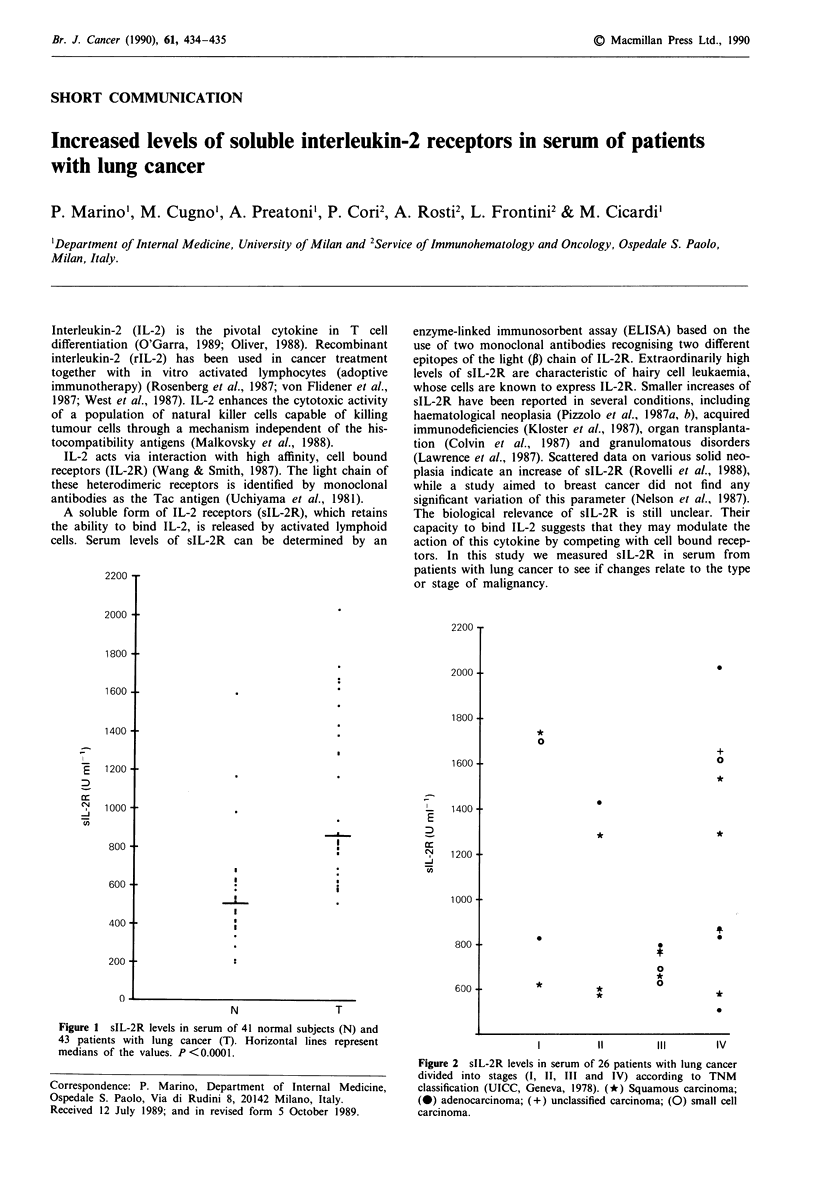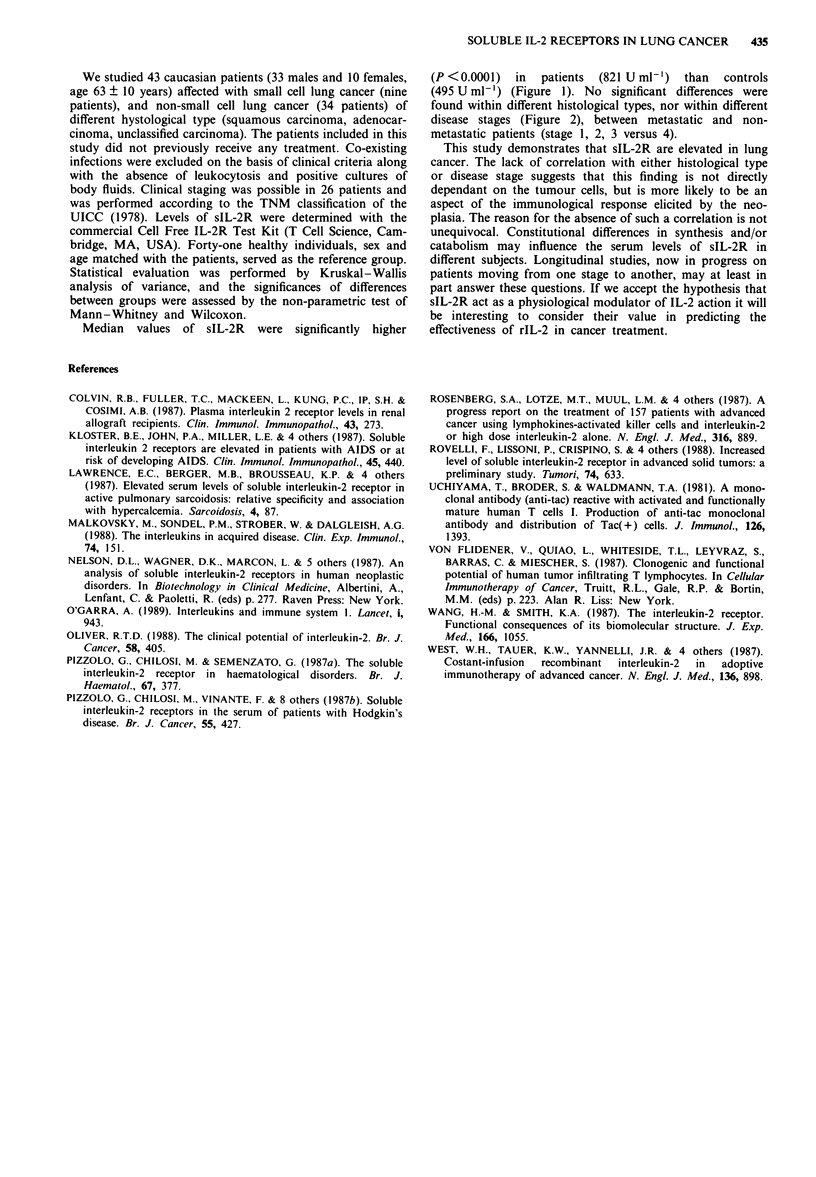# Increased levels of soluble interleukin-2 receptors in serum of patients with lung cancer.

**DOI:** 10.1038/bjc.1990.95

**Published:** 1990-03

**Authors:** P. Marino, M. Cugno, A. Preatoni, P. Cori, A. Rosti, L. Frontini, M. Cicardi

**Affiliations:** Department of Internal Medicine, University of Milan, Italy.


Br. J. Cancer (1990), 61, 434-435 © Macmillan Press Ltd., 1990

SHORT COMMUNICATION

### Increased levels of soluble interleukin-2 receptors in serum of patients with lung cancer

P. Marino[1], M. Cugno[1], A. Preatoni[1], P. Cori[2], A. Rosti[2], L. Frontini[2] & M. Cicardi[1]

[1]*Department of Internal Medicine, University of Milan and* [2]*Service of Immunohematology and Oncology, Ospedale S. Paolo, Milan, Italy.*

Interleukin-2 (IL-2) is the pivotal cytokine in T cell differentiation (O'Garra, 1989; Oliver, 1988). Recombinant interleukin-2 (rIL-2) has been used in cancer treatment together with in vitro activated lymphocytes (adoptive immunotherapy) (Rosenberg *et al.*, 1987; von Flidener *et al.*, 1987; West *et al.*, 1987). IL-2 enhances the cytotoxic activity of a population of natural killer cells capable of killing tumour cells through a mechanism independent of the histocompatibility antigens (Malkovsky *et al.*, 1988).

IL-2 acts via interaction with high affinity, cell bound receptors (IL-2R) (Wang & Smith, 1987). The light chain of these heterodimeric receptors is identified by monoclonal antibodies as the Tac antigen (Uchiyama *et al.*, 1981).

A soluble form of IL-2 receptors (sIL-2R), which retains the ability to bind IL-2, is released by activated lymphoid cells. Serum levels of sIL-2R can be determined by an enzyme-linked immunosorbent assay (ELISA) based on the use of two monoclonal antibodies recognising two different epitopes of the light (β) chain of IL-2R. Extraordinarily high levels of sIL-2R are characteristic of hairy cell leukaemia, whose cells are known to express IL-2R. Smaller increases of sIL-2R have been reported in several conditions, including haematological neoplasia (Pizzolo *et al.*, 1987*a*, *b*), acquired immunodeficiencies (Kloster *et al.*, 1987), organ transplantation (Colvin *et al.*, 1987) and granulomatous disorders (Lawrence *et al.*, 1987). Scattered data on various solid neoplasia indicate an increase of sIL-2R (Rovelli *et al.*, 1988), while a study aimed to breast cancer did not find any significant variation of this parameter (Nelson *et al.*, 1987). The biological relevance of sIL-2R is still unclear. Their capacity to bind IL-2 suggests that they may modulate the action of this cytokine by competing with cell bound receptors. In this study we measured sIL-2R in serum from patients with lung cancer to see if changes relate to the type or stage of malignancy.

**Figure 1** sIL-2R levels in serum of 41 normal subjects (N) and 43 patients with lung cancer (T). Horizontal lines represent medians of the values. $P < 0.0001$.

**Figure 2** sIL-2R levels in serum of 26 patients with lung cancer divided into stages (I, II, III and IV) according to TNM classification (UICC, Geneva, 1978). (★) Squamous carcinoma; (●) adenocarcinoma; (+) unclassified carcinoma; (O) small cell carcinoma.

Correspondence: P. Marino, Department of Internal Medicine, Ospedale S. Paolo, Via di Rudini 8, 20142 Milano, Italy.
Received 12 July 1989; and in revised form 5 October 1989.

We studied 43 caucasian patients (33 males and 10 females, age 63 ± 10 years) affected with small cell lung cancer (nine patients), and non-small cell lung cancer (34 patients) of different hystological type (squamous carcinoma, adenocarcinoma, unclassified carcinoma). The patients included in this study did not previously receive any treatment. Co-existing infections were excluded on the basis of clinical criteria along with the absence of leukocytosis and positive cultures of body fluids. Clinical staging was possible in 26 patients and was performed according to the TNM classification of the UICC (1978). Levels of sIL-2R were determined with the commercial Cell Free IL-2R Test Kit (T Cell Science, Cambridge, MA, USA). Forty-one healthy individuals, sex and age matched with the patients, served as the reference group. Statistical evaluation was performed by Kruskal–Wallis analysis of variance, and the significances of differences between groups were assessed by the non-parametric test of Mann–Whitney and Wilcoxon.

Median values of sIL-2R were significantly higher ($P < 0.0001$) in patients ($821\ \mathrm{U\ ml^{-1}}$) than controls ($495\ \mathrm{U\ ml^{-1}}$) (Figure 1). No significant differences were found within different histological types, nor within different disease stages (Figure 2), between metastatic and non-metastatic patients (stage 1, 2, 3 versus 4).

This study demonstrates that sIL-2R are elevated in lung cancer. The lack of correlation with either histological type or disease stage suggests that this finding is not directly dependant on the tumour cells, but is more likely to be an aspect of the immunological response elicited by the neoplasia. The reason for the absence of such a correlation is not unequivocal. Constitutional differences in synthesis and/or catabolism may influence the serum levels of sIL-2R in different subjects. Longitudinal studies, now in progress on patients moving from one stage to another, may at least in part answer these questions. If we accept the hypothesis that sIL-2R act as a physiological modulator of IL-2 action it will be interesting to consider their value in predicting the effectiveness of rIL-2 in cancer treatment.

### References

COLVIN, R.B., FULLER, T.C., MACKEEN, L., KUNG, P.C., IP, S.H. & COSIMI, A.B. (1987). Plasma interleukin 2 receptor levels in renal allograft recipients. *Clin. Immunol. Immunopathol.*, **43,** 273.

KLOSTER, B.E., JOHN, P.A., MILLER, L.E. & 4 others (1987). Soluble interleukin 2 receptors are elevated in patients with AIDS or at risk of developing AIDS. *Clin. Immunol. Immunopathol.*, **45,** 440.

LAWRENCE, E.C., BERGER, M.B., BROUSSEAU, K.P. & 4 others (1987). Elevated serum levels of soluble interleukin-2 receptor in active pulmonary sarcoidosis: relative specificity and association with hypercalcemia. *Sarcoidosis*, **4,** 87.

MALKOVSKY, M., SONDEL, P.M., STROBER, W. & DALGLEISH, A.G. (1988). The interleukins in acquired disease. *Clin. Exp. Immunol.*, **74,** 151.

NELSON, D.L., WAGNER, D.K., MARCON, L. & 5 others (1987). An analysis of soluble interleukin-2 receptors in human neoplastic disorders. In *Biotechnology in Clinical Medicine*, Albertini, A., Lenfant, C. & Paoletti, R. (eds) p. 277. Raven Press: New York.

O'GARRA, A. (1989). Interleukins and immune system 1. *Lancet*, **i,** 943.

OLIVER, R.T.D. (1988). The clinical potential of interleukin-2. *Br. J. Cancer*, **58,** 405.

PIZZOLO, G., CHILOSI, M. & SEMENZATO, G. (1987*a*). The soluble interleukin-2 receptor in haematological disorders. *Br. J. Haematol.*, **67,** 377.

PIZZOLO, G., CHILOSI, M., VINANTE, F. & 8 others (1987*b*). Soluble interleukin-2 receptors in the serum of patients with Hodgkin's disease. *Br. J. Cancer*, **55,** 427.

ROSENBERG, S.A., LOTZE, M.T., MUUL, L.M. & 4 others (1987). A progress report on the treatment of 157 patients with advanced cancer using lymphokines-activated killer cells and interleukin-2 or high dose interleukin-2 alone. *N. Engl. J. Med.*, **316,** 889.

ROVELLI, F., LISSONI, P., CRISPINO, S. & 4 others (1988). Increased level of soluble interleukin-2 receptor in advanced solid tumors: a preliminary study. *Tumori*, **74,** 633.

UCHIYAMA, T., BRODER, S. & WALDMANN, T.A. (1981). A monoclonal antibody (anti-tac) reactive with activated and functionally mature human T cells I. Production of anti-tac monoclonal antibody and distribution of Tac(+) cells. *J. Immunol.*, **126,** 1393.

VON FLIDENER, V., QUIAO, L., WHITESIDE, T.L., LEYVRAZ, S., BARRAS, C. & MIESCHER, S. (1987). Clonogenic and functional potential of human tumor infiltrating T lymphocytes. In *Cellular Immunotherapy of Cancer*, Truitt, R.L., Gale, R.P. & Bortin, M.M. (eds) p. 223. Alan R. Liss: New York.

WANG, H.-M. & SMITH, K.A. (1987). The interleukin-2 receptor. Functional consequences of its biomolecular structure. *J. Exp. Med.*, **166,** 1055.

WEST, W.H., TAUER, K.W., YANNELLI, J.R. & 4 others (1987). Costant-infusion recombinant interleukin-2 in adoptive immunotherapy of advanced cancer. *N. Engl. J. Med.*, **136,** 898.